# Integrated Hair Follicle Profiles of microRNAs and mRNAs to Reveal the Pattern Formation of Hu Sheep Lambskin

**DOI:** 10.3390/genes13020342

**Published:** 2022-02-14

**Authors:** Xiaoyang Lv, Weihao Chen, Shanhe Wang, Xiukai Cao, Zehu Yuan, Tesfaye Getachew, Joram M. Mwacharo, Aynalem Haile, Wei Sun

**Affiliations:** 1 Joint International Research Laboratory of Agriculture and Agri-Product Safety of Ministry of Education of China, Yangzhou University, Yangzhou 225009, China; dx120170085@yzu.edu.cn (X.L.); cxkai0909@163.com (X.C.); yuanzehu1988@163.com (Z.Y.); 2 College of Animal Science and Technology, Yangzhou University, Yangzhou 225009, China; 18552133709@163.com (W.C.); shanhe12315@163.com (S.W.); 3 International Centre for Agricultural Research in the Dry Areas, Addis Ababa 999047, Ethiopia; t.getachew@cgiar.org (T.G.); j.mwacharo@cgiar.org (J.M.M.); a.haile@cgiar.org (A.H.)

**Keywords:** Hu sheep, hair follicle, miRNA, mRNA, wool curvature

## Abstract

Hair follicle development is closely associated with wool curvature. Current studies reveal the crucial role of microRNAs (miRNAs) in hair follicle growth and development. However, few studies are known regarding their role in wool curvature. To reveal the potential roles of miRNAs in Hu sheep lambskin with different patterns, a total of 37 differentially expressed (DE) miRNAs were identified in hair follicles between small waves (SM) and straight wool (ST) groups using RNA-seq. Through functional enrichment and miRNA-mRNA co-expression analysis, some key miRNAs (oar-miR-143, oar-miR-200b, oar-miR-10a, oar-miR-181a, oar-miR-10b, oar-miR-125b, etc.) and miRNA-mRNA pairs (miR-125b target *CD34*, miR-181a target *FGF12*, *LMO3*, miR-200b target *ZNF536*, etc.) were identified. Though direct or indirect ways affecting hair follicle development, these miRNAs and mRNAs may have possible effects on wool curvature, and this study thus provides valuable insight on potential pattern formation.

## 1. Introduction

Hu sheep is a unique species of lambskin in China, characterized by precocity and high productivity. It is well known for its gentle lambskin and beautiful water-wave pattern. Hu lambskin thus holds economic value because of its popularity among certain consumers and its quality is based on the visible wave pattern. The wave pattern determines the quality of lambskin, and the quality of small waves is excellent, while straight wool is poor. The type of wave pattern is determined by many factors, such as curvature, density, and fineness of the wool. Wool curvature is the key factor of the wave pattern of lambskin, which is decided by hair follicle growth and development [1]. Hair follicle growth and development is a complex process, which participated in delicate molecular regulatory mechanisms. Although its genetic basis in French breeds has recently been unraveled as an insertion/deletion polymorphism (InDel) in the 3′UTR part of the IRF2BP2 gene [2], it remains unclear whether the same mechanism exists for Hu sheep. If so, a Hu sheep population of high-quality lambskin might be bred via molecular breeding, thus allowing for the conservation of the Hu sheep lambskin trait and increasing the production of high-quality lambskin.

Recently, many reports have indicated that multiple genes may be involved in hair follicle growth and development, such as *Wnt10a* [3], *lef1* [4], *Sox9* [5], *BMP4* [6]. Additionally, some signaling pathways, such as BMP [7], Eda [8], Shh [9], and TGF-β [10] signaling, were revealed to promote or suppress the process of hair follicle growth and development. With the advancement of genomics, environment–gene interactions have become a research hotspot, and epistatic regulation may represent a bridge between the environment and genetics. Studies have shown that rearing cashmere goats under controlled lighting conditions may enhance cashmere production during non-cashmere seasons. It was also suggested that these goats may have acquired epigenetic memory, as they continued with this production pattern in the following year (after experiments have ceased). Wu et al. noted a significant increase in melatonin-related *Hoxc13* expression which also positively triggered hair follicle development-relevant genes. It is, however, known that melatonin changes seasonally and environmental factors may thus influence genetic factors that are involved in hair follicles’ development [11].

MicroRNAs (miRNAs) are small endogenous RNAs (consisting of approximately 22 nucleotides) that are considered to play important regulatory roles in biological processes by targeting mRNAs or repressing translation [12]. In addition to being associated with many diseases, studies have linked miRNAs to the regulation of cell development, proliferation, and apoptosis, especially associated with many diseases [13]. With the further research of miRNA, the hair follicle growth and development process may also be controlled by epigenetic regulator factor miRNAs, except for multiple genes and signaling pathways. MiRNA and its target gene form diverse regulatory networks, playing an important role in hair follicle growth and development. Current research has found that miRNAs are expressed in a variety of different hair follicle cells, such as hair follicle stem cells, matrix cells, outer root sheath cells, inner root sheath cells, and lots of miRNAs are verified to be specifically expressed in hair follicle cells [14]. For example, both miR-125b [15] and miR-205 [16] are expressed in hair follicle stem cells, but they have the opposite effect. MiR-125b may act as a repressor to suppress stem cell differentiation, while miR-205 has a positive effect on stem cells and the proliferation of their progenies. Similarly, miR-214 [17] and miR-31 [18] are expressed in the outer root sheath and hair follicle matrix cells where they induce different hair follicle functions. MiR-24 is richly expressed in the inner root sheath and competitively binds to *TCF-3* to promote hair follicle keratinocyte differentiation. However, it is unclear in which cells miR-137 is richly expressed, but miR-137 is associated with coat color.

MiRNA is normally about 20–25 nucleotides with a seed sequence of 6–8 nucleotides. Various studies have shown that miRNAs could competitively bind to 3′UTR of mRNAs to affect the expression of the target genes [19]. Of course, the targeting relationship between miRNA and mRNA is not one by one. Years of research have indicated that the targeting relationship between miRNA and mRNA is not one-on-one, but that miRNA may regulate the expression of multiple or even hundreds of genes [20]. For example, a previous study on the changes in hair follicle morphogenesis indicated that the latter had been related to the expression of miR-125b and around 800 transcripts with which miR-125b may be targeted [15]. Similarly, miR-205 has been identified to competitively bind to 3′UTR of *INPP1b*, *INPP4b*, *PHLDA2*, and *FRK*, whereas miR-31 also has four validated target genes, *KRT16*, *KRT17*, *FGF10*, and *DLX3* [14]. It has also been suggested that, under certain circumstances, the biological function of miRNAs may be predicted through their target genes.

The keratin (KRT) family is an important components of hair follicle. More specifically, *KRT27*, *KRT35*, and *KRT85* were found to be involved in the construction of the initially differentiated cortical structure, which ultimately leads to fibrillar bending [21], while, *KRT17* was involved in the regulation of the hair follicle cycle and acted as a positive regulator of hair follicle growth [22]. Since miRNAs can target genes to regulate traits, we hypothesize that when either miRNAs or target genes are associated with hair follicle development, it is highly likely that the corresponding miRNAs or target genes might also be involved in the same process. For example, since *KRT17* is involved in the hair follicle cycle, then miR-31 may be a candidate miRNA to investigate its role in hair follicle development.

Much work has been performed on the hair follicles of Hu sheep and we previously reported a total of 114 DE circular RNA (cirRNAs) [23] and 72 DE long non-coding RNAs (lncRNAs) [24] between small waves and straight wool after completing whole transcriptome sequencing. In the same study, 137 DE mRNAs between large waves and small waves, and *BMP7*, *MMP2*, *SNAI1*, *CDKNIC*, *MT3*, and *POU1F1* were selected to act as candidate genes [25]. Furthermore, to distinguish between small, medium, and large waves, miRNA sequencing was performed and DE-miRNAs (miR-143, miR-10a, let-7a, etc.) were screened association analysis with hair follicle metrics (diameters and numbers) [26]. Since the consideration of small differences between the three pattern types, the DE miRNAs were not accurate enough, so this experiment further mined DE miRNAs from straight wool and small waves.

In summary, miRNA plays an extremely important role in hair follicle development. By thoroughly screening the DE miRNA and its target genes between small waves and straight wool of hair follicle in Hu sheep lambskin. It may provide the basis for further explorations. Specifically, understanding miRNA regulation in the formation of different patterns in Hu sheep lambskin might allow for the breeding of new Hu sheep strains of with high-quality lambskin.

## 2. Materials and Methods

### 2.1. Sample Collection

Animal research proposals were approved by the Animal Care and Use Committee at Yangzhou University. All animal experimental procedures were implemented in strict obedience of the guidelines for the Animal Care and Use Committee at Yangzhou University to minimize the suffering of animals.

**Figure 1:**
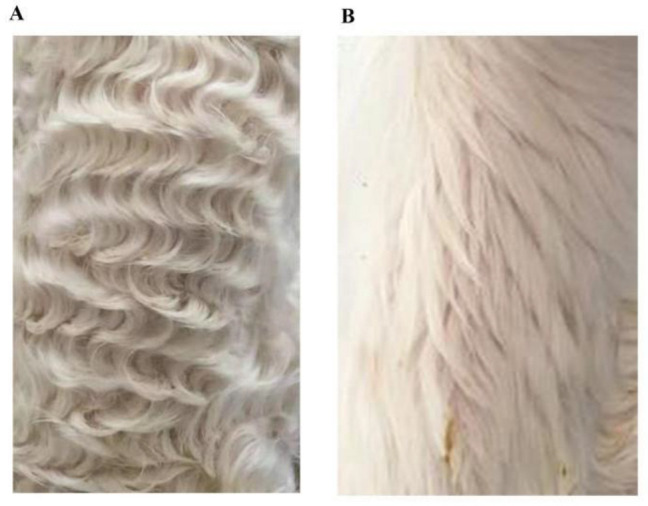
Identification of two pattern phenotype in Hu sheep. (**A**) Small waves. (**B**) Straight wool.

A total of 6 two-day-old healthy Hu lambs were selected from the Suzhou Stud Farm in Jiangsu Province, China. Three pairs of full-sib individuals were divided into small waves groups (SM1, SM2, SM3) and straight wool groups (ST1, ST2, ST3) with three replicates (Figure 1). The phenotype of Lambskin was classified as small waves and straight wool according to the width of the pattern that is the length between the bulges at the ends of a single pattern. A pattern with a width of less than 1.25 cm was deemed a “small” pattern, whereas wool that barely curved was deemed as “straight wool”. Approximately 1 cm of hair root was cut off from the dorsal side of Hu lambs. A total of 1/3 volume of the tube was collected and stored in Drikold.

### 2.2. Library Preparation for Sequencing

Total RNA was extracted from the hair roots of small waves and straight wool groups using RNAiso Plus Kit (Takara, Kusatsu, Shiga, Japan) according to the manufacturer’s instructions. After total RNA isolation, the quality and concentration of RNA were measured to meet the sequencing requirements. The mRNA libraries were generated with 3 μg high-quality total RNA per sample through NEBNext^®^ UltraTM RNA Library Prep Kit Illumina^®^ (NEB, Ipswich, MA, USA) following the manufacturer’s recommendations. Firstly, mRNA was purified with ploy-T oligo-attached magnetic beads. First-strand cDNA synthesized by M-MuLV Reverse Transcriptase and random hexamer primer. Then, RNase H and polymerase I were used to synthesize the second cDNA. The cDNA fragments of 150–200 bp were selected by AMPure XP system (Beckman Coulter, Beverly, MA, USA) and ligated to NEBNext Adaptor. The quality of PCR product was assessed on the Agilent Bioanalyzer 2100 system. The library was sequenced (150 bp paired-end) using Illumina Hiseq 2500 platform.

Simultaneously, a total of 3 μg total RNA per sample was applied to construct the small RNA library through NEBNext^®^ Multiplex Small RNA Library Prep Set for Illumina^®^ (NEB, Ipswich, MA, USA) following the manufacturer’s recommendations. The 3′ ends and 5′ ends adaptor were directly and specifically ligated to the 3′ end and 5′ ends of miRNA, siRNA, and piRNA. Then, the first-strand of cDNA was synthesized using M-MuLV Reverse Transcriptase, and PCR amplification was implemented. DNA fragments corresponding to 140–160 bp were recovered and dissolved in the elution buffer. After the cDNA library was assessed on the Agilent Bioanalyzer 2100 system using DNA High Sensitivity Chips, the libraries were sequenced on an Illumina Hiseq 2500 platform (50 bp single-end).

### 2.3. Quality Control, Reference Genome Alignment, and Assembly

Raw reads were obtained from sequencing and the quality control was carried out through custom perl and python scripts. Clean reads were purified by removing low-quality reads and reads containing ploy-N, polyA/T/G/C, with 5′ adapter contaminants, without 3′ adapter or the insert tag. Subsequently, error rates, Q20, Q30, and GC content were calculated [1,27]. Then, clean reads were used for all the downstream analyses. The reference genome (Ovis aries v4.0) and gene annotation files were downloaded from the genome website (http://www.ensembl.org/index.html, accessed on 9 May 2021). Hisat2 (http://ccb.jhu.edu/software/hisat2, accessed on 9 May 2021) and Stringtie (http://ccb.jhu.edu/software/stringtie/, accessed on 9 May 2021) were used to map mRNA to reference sequence and to splice the transcript.

For miRNAs, the reads mapped to reference sequence were compared with the sequences in the specified range in miRBase [28]. To remove tags formed by rRNA, tRNA, snRNA, snoRNA, repeat sequences, and mRNA, small RNA tags were mapped to Rfam database and RepeatMasker. At the same time, miREvo [29] and mirdeep2 software [30] were integrated to predict novel miRNA through exploring the minimum free energy, Dicer cleavage site, and secondary structure of the unannotated small RNA tags, the miRNA first nucleotide bias with different lengths and miRNA nucleotide bias at each position, respectively. According to the priority rule: known miRNA > rRNA > tRNA > snRNA > snoRNA > repeat > novel miRNA; each small RNA was mapped to only one annotation.

### 2.4. Quantification and Differential Expression of miRNA and mRNA

In this experiment, miRNA and mRNA expression levels were estimated through transcript per million (TPM) and fragments per kilobase per million mapped reads (FPKM) [31], respectively. To ensure the accuracy of the subsequent screening of DE miRNAs, we firstly carried out Pearson correlation analysis of samples.

Due to small waves and straight wool groups with three biological replicates, DESeq2 R package (1.8.1) was used for differential expression analysis of these two groups. Flog change and corrected *p*-values that were adjusted using the Benjamini and Hochberg approach were used to assess the significant difference of miRNAs and mRNAs expression levels in different groups. Corrected *p*-values < 0.05 were considered to be significantly differentially expressed [32]. Additionally, volcano plots and heat maps were adopted to screen differentially expressed miRNAs and mRNAs.

### 2.5. Target Gene Prediction and Functional Enrichment Analysis of miRNAs

Predicting the target gene of known and novel miRNA was performed by miRanda and RNAhybrid software for straight wool and small waves groups [33]. The correspondence relationship between miRNA and target gene was obtained through the intersection of predicted results.

According to the results of predicting the target gene of DE miRNA, Gene Ontology (GO) and Kyoto Encyclopedia of Genes and Genomes (KEGG) pathway enrichment analysis were used on the target gene candidates of differentially expressed miRNAs. GO enrichment analysis was implemented using the GOseq method based on Wallenius non-central hyper-geometric distribution [34]. KEGG database resource is a tool for understanding high-level functions and utilities of the biological system. The statistical enrichment of the target gene candidates was estimated by KOBAS software [35]. The clusterProfiler R package was applied to analyze GO and KEGG pathway enrichment of the target gene candidates. It (*p* < 0.05) was considered significantly enriched.

### 2.6. Integrated Analysis of miRNA and mRNA Expression Profiles

To further analyze the relationship between DE miRNAs and DE mRNAs related to hair follicle development, a network of miRNA-mRNA needed to be constructed based on their expression levels and functions through Cytoscape_2.8.3 software.

### 2.7. Verification of RNA-seq Results

To verify the reliability of the RNA-seq results, 6 DE miRNAs and 6 DE mRNAs were randomly selected with U6 and *GAPDH* as reference genes, respectively. The detailed information on relevant primers is shown in Supplementary Table S1. Each sample with three replicates was tested to establish a standard curve. The Real-time Quantitative Polymerase Chain Reaction (RT-qPCR) amplification was performed under the following procedure: initial denaturation at 95 °C for 5 min, circular reaction at 95 °C for 10 s, and 60 °C for 30 s with 40 cycles. The relative expression levels of DE miRNAs and mRNAs were calculated by 2^−ΔΔCt^ method. A dependent sample *t*-test was performed to calculate relative expression and SPSS13.0 was used to analyze the significant difference. All the results were shown as mean ± SD.

## 3. Results

### 3.1. The Expression Profile of miRNA and mRNA

**Figure 2:**
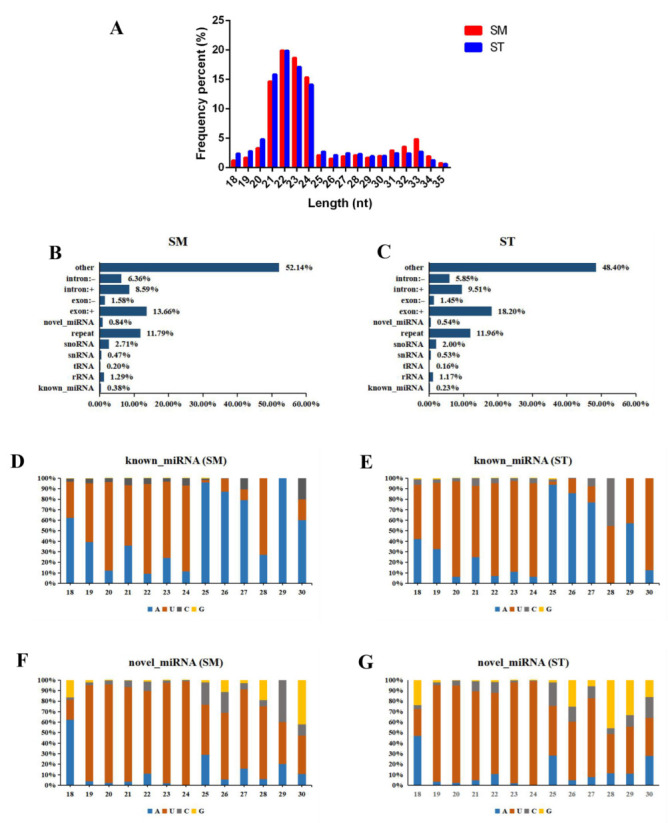
Identification and characterization of miRNA. (**A**) Length distribution of total sRNA fragment. (**B**) Classified identification of all sRNA in small waves. (**C**) Classified identification of all sRNA in straight wool. (**D**) First base preference analysis of known miRNA in small waves. (**E**) First base preference analysis of known miRNA in straight wool. (**F**) First base preference analysis of novel miRNA in small waves. (**G**) First base preference analysis of novel miRNA in straight wool.

The expression profiles of miRNAs and mRNAs were investigated in hair follicles between straight wool and small waves using RNA-seq. Regarding miRNA, a total of approximately 32,724,357 and 38,996,977 raw reads were generated in SM and ST, respectively, and 31,173,567 and 37,529,529 clean reads were filtered for subsequent analysis by RNA-seq (Supplementary Table S2). The length interval of sRNA was 18 to 35 nt by length distribution statistics, and miRNAs were concentrated in 21 to 22 nt (Figure 2A). Additionally, miRNAs were distributed on chromosome 1 to X (Supplementary Figure S1), and most miRNAs were located on the chromosome 1, 2, 3. Moreover, known miRNA, novel miRNA, tRNA, rRNA, etc. were identified by alignment and annotation, but the known miRNA and novel miRNA account for only a small fraction of non-coding RNA (Figure 2B,C). Base preference analysis of miRNAs with different lengths showed that first base distribution of the miRNAs with 20 to 24 nt were mainly U (Figure 2D–G), which were consistent with the characteristics of miRNA.

**Figure 3:**
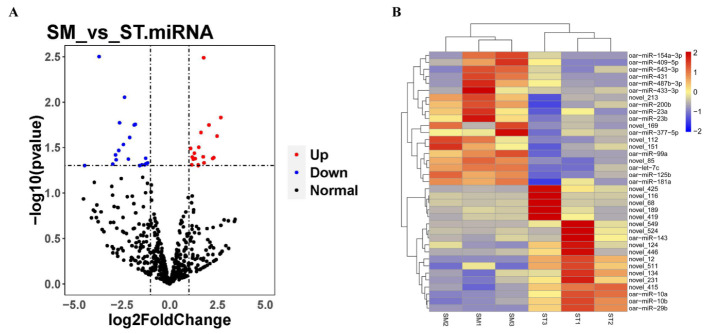
Significant difference analysis of miRNAs. (**A**) Volcano plot of identified miRNAs between SM vs. ST, where blue and red represent down-regulated and up-regulated, respectively. (**B**) Heat maps revealing the differentially expressed (DE) miRNA in hair follicle between SM vs. ST.

According to the results of Pearson correlation analysis between samples, the correlation coefficient between biological replicates was higher in the small waves group, and the expression pattern was similar, as well as in the straight wool group (Supplementary Figure S2). According to the TPM of miRNAs estimating expression levels, 595 miRNAs were identified in hair follicles between SM vs. ST, containing 37 DE miRNAs (18 known miRNAs and 19 novel miRNAs) consisting of 18 up-regulated and 19 down-regulated miRNAs (Figure 3A and Supplementary Table S3). Furthermore, the expression profile of DE miRNAs was obviously presented to different expression patterns in Figure 3B.

**Figure 4:**
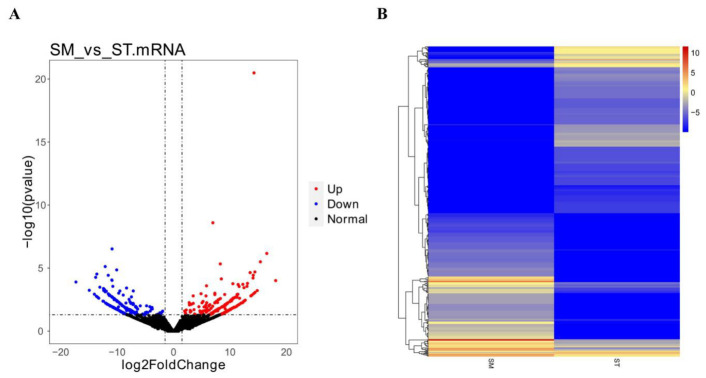
Significant difference analysis of mRNAs. (**A**) Volcano plot of identified mRNAs between SM vs. ST, where blue and red represent down-regulated and up-regulated, respectively. (**B**) Heat maps revealing the differentially expressed (DE) mRNA in hair follicle between SM vs. ST.

Regarding mRNA, a total of approximately 330,913,612 and 296,509,262 raw reads were generated in SM and ST, respectively, and 325,445,988 and 290,032,082 clean reads were filtered for subsequent analysis (Supplementary Table S4). According to the FPKM of mRNAs estimating gene expression levels, 17,786 mRNAs were identified in hair follicle, suggesting that these data were reasonable. The mRNA difference significance analysis showed that a total of 489 DE mRNAs were identified between SM vs. ST (Figure 4A and Supplementary Table S5), of which 222 were up-regulated and 267 down-regulated DE mRNAs between SM vs. ST. Additionally, the expression profile of DE mRNAs was obviously presented to different expression patterns in Figure 4B.

### 3.2. Functional Enrichment Analysis of Target Genes

**Figure 5:**
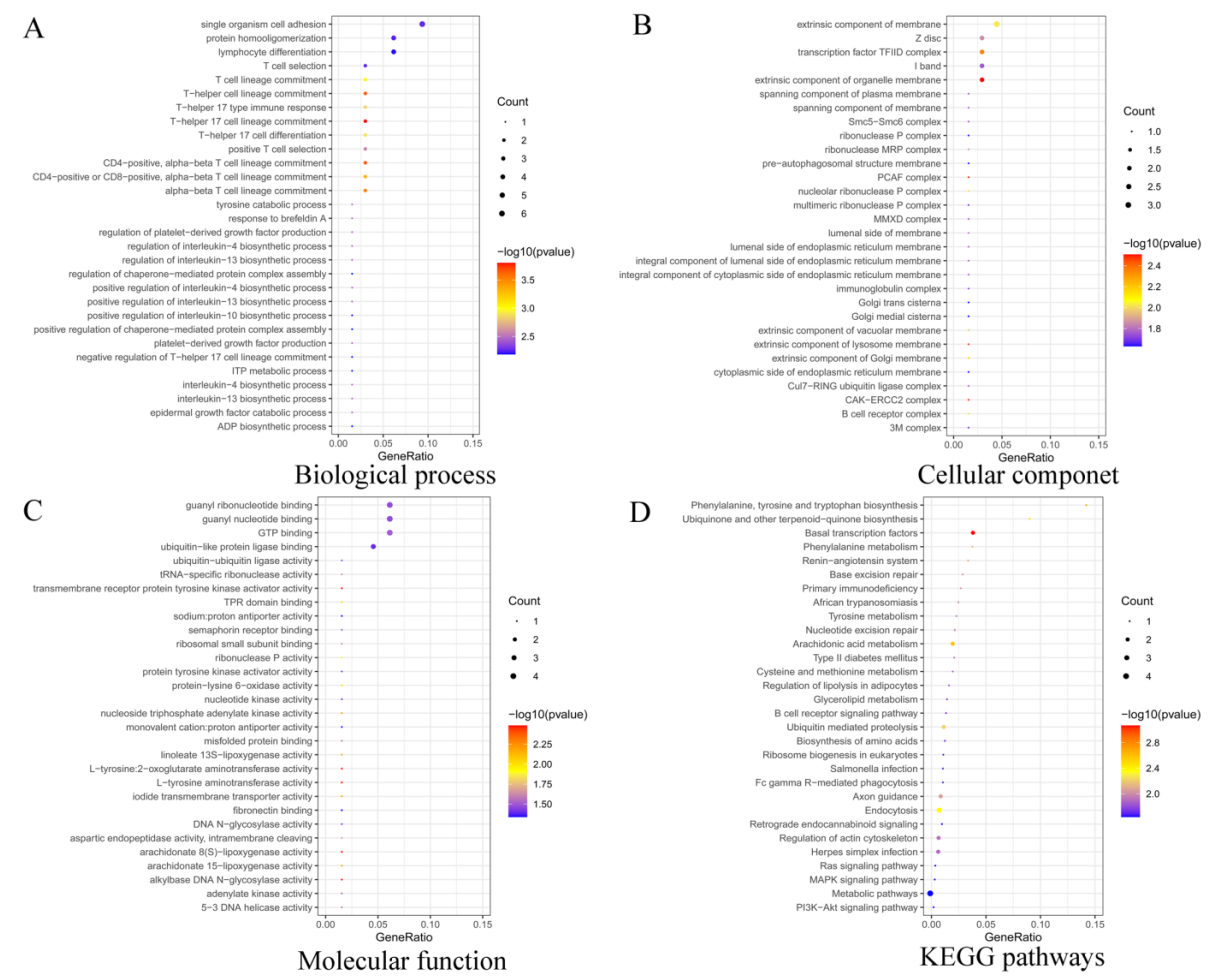
GO and KEGG functional enrichment analysis of target genes. (**A**) Top 30 GO term with biological process in SM vs. ST. (**B**) Top 30 GO term with cellular component in SM vs. ST. (**C**) Top 30 GO term with molecular function in SM vs. ST. (**D**) Top 30 KEGG pathways in SM vs. ST.

To better understand the biological functions of DE miRNAs, the Gene Ontology (GO) and KEGG pathway analysis were performed on their target genes using the target gene prediction method. The results showed that 37 DE miRNAs corresponded to 533 target genes, and GO enrichment analysis of target genes, a total of 249 GO terms were significantly enriched, containing biological process (BP), cellular component (CC), and molecular function (MF) (Supplementary Table S6). From Figure 4, the top enriched GO term is intracellular organelle (GO:0043229), cellular component organization or biogenesis (GO:0071840), and GTP binding (GO:0005525) in CC, BP, and MF, respectively. Moreover, some GO terms, such as epithelial cell differentiation (GO:0030855), regulation of epithelial cell differentiation (GO:0030856), and epidermal growth factor catabolic process (GO:0007174) in BP, suggest the crucial role in epithelial cell proliferation and differentiation, to a certain extent, an effect on hair growth. Some GO terms related to the preparation of transcription include DNA-directed RNA polymerase II, holoenzyme (GO:0016591) and nuclear DNA-directed RNA polymerase complex (GO:0055029) in CC. Some GO terms involved in energy supply and enzyme synthesis include nucleoside triphosphate adenylate kinase activity (GO:0046899) and tRNA-specific ribonuclease activity (GO:0004549) in MF (Figure 5A–C and Supplementary Table S6).

KEGG pathway analysis revealed that these target genes were enriched in 52 KEGG terms, with the most enriched pathway being that of metabolic pathways (oas01100) (Figure 5D and Supplementary Table S7). Specifically, Ras signaling pathway (oas04014), MAPK signaling pathway (oas04010), and PI3K-Akt signaling pathway (oas04151) were found, which are known to be associated with hair follicle development [36,37,38].

### 3.3. Co-Expression Analysis of Integrated DE miRNA-mRNA

**Figure 6:**
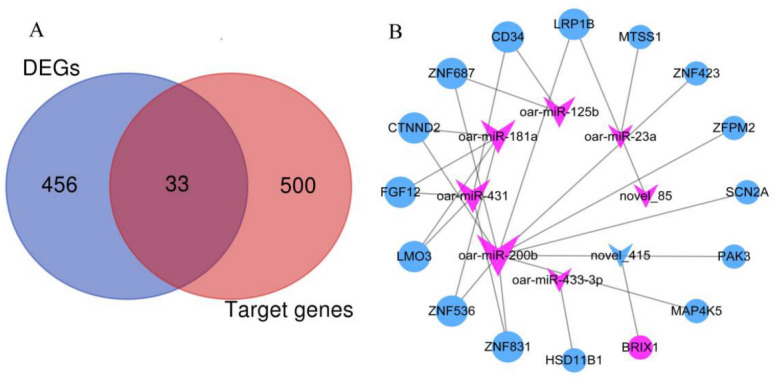
Co-expression network of miRNA-mRNA. (**A**) Overlapped genes between DE genes and target genes. (**B**) The interaction network of miRNAs and overlapped genes in SM vs. ST. “v” sharp represents DE miRNAs and circle represents overlapped genes. Red represents up-regulated miRNAs or genes and blue represents down-regulated miRNAs or genes.

To further understand the potential role of miRNAs in hair follicle development, an interaction network was constructed using DE miRNAs and mRNAs. In fact, target gene prediction of 37 DE miRNAs was performed to target 533 genes (Supplementary Table S8). Furthermore, we performed intersection analysis of 489 DE mRNAs and 533 predicted target genes; 33 overlapped genes were obtained (Figure 6A). Based on the principle of opposite miRNA and mRNA expression levels, a miRNA-mRNA co-expression network was constructed, where 8 DE miRNAs were targeted to 16 genes. The co-expression network revealed that 7 up-regulated miRNAs targeted 15 down-regulated genes, while 1 down-regulated miRNA targeted 1 up-regulated gene (Figure 6B).

### 3.4. Data Validation

**Figure 7:**
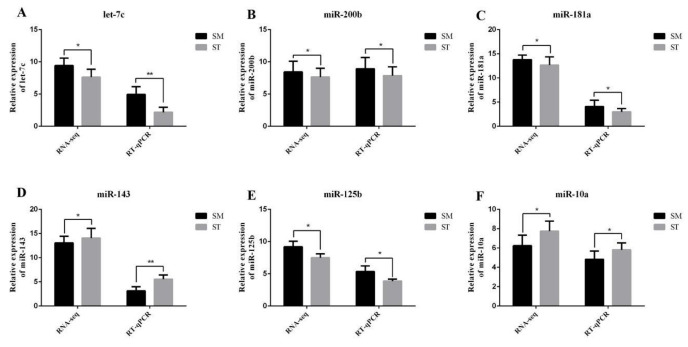
Verification of miRNAs sequencing by RT-qPCR between SM and ST. (**A**) Relative expression of let-7c. (**B**) Relative expression of miR-200b. (**C**) Relative expression of miR-181a. (**D**) Relative expression of miR-143. (**E**) Relative expression of miR-125b. (**F**) Relative expression of miR-10a. * represents statistical significance (*p* < 0.05) and ** represents extremely significant difference (*p* < 0.01).

**Figure 8:**
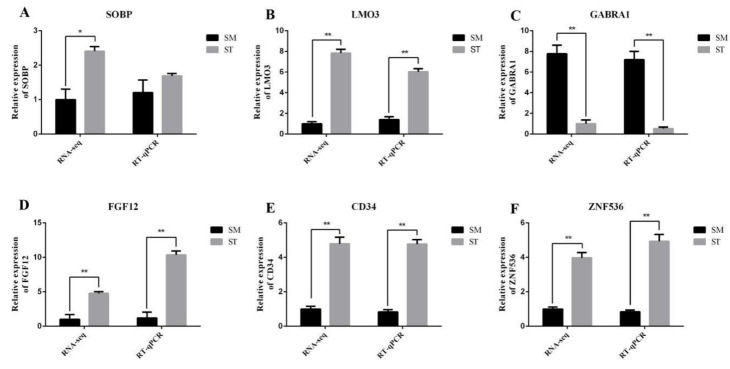
Verification of mRNAs sequencing by RT-qPCR between SM and ST. (**A**) Relative expression of SOBP mRNA. (**B**) Relative expression of LMO3 mRNA. (**C**) Relative expression of GABRA1 mRNA. (**D**) Relative expression of FGF12 mRNA. (**E**) Relative expression of CD34 mRNA. (**F**) Relative expression of ZNF536 mRNA. * represents statistical significance (*p* < 0.05) and ** represents extremely significant difference (*p* < 0.01).

To verify the accuracy of RNA-seq, RT-qPCR was performed. The latter showed similar expression patterns for both miRNAs and mRNAs to their respective RNA-seq results, which suggests reliability in the sequencing (Figure 6). In addition, the expression patterns of miR-125b and *CD34*, miR-181a and *FGF12*, *LMO3*, miR-200b, and *ZNF536* displayed an opposite trend in SM and ST, coinciding with the miRNA-mRNA co-expression network result (Figure 7 and Figure 8).

## 4. Discussion

### 4.1. miRNAs and Hair Follicle Development

Hair follicle development is controlled by the changes of multiple cellular activities in the hair follicle which, in turn, are regulated by various transcription factors, signaling pathways, and epigenetic regulation. Among them, miRNA-mRNA regulatory networks participate in hair follicle cellular activity and thereby influence hair follicle development [14]. Some studies have suggested a tight association between the latter and wool curvature or, more specifically, that dermal papilla and other hair follicle cell activities are responsible for wool curvature via direct or indirect ways [39]. By far, the studies show that a lack of the miRNA processors *Dier* may lead to abnormal development, suggesting the indispensable role of miRNAs in hair follicle growth and development [40]. Previously, miR-125b and miR-205 were confirmed to respectively act as an activator and repressor of hair follicle stem cell differentiation [15,16], while miR-214 and miR-31 have been found to be highly expressed in proliferation hair matrix cells [17,18]. As such, it is not only necessary but also feasible to study hair follicle development from a miRNA perspective.

### 4.2. DE miRNAs Analysis in Different Degree of Curliness

In a previous study, we identified 13 DE-miRNAs between small and large wave patterns in Hu sheep [41]. In the current study, as more DE-miRNAs were screened (seeing that the difference between SM and ST was greater than that between small and large waves), a total of 37 DE-miRNAs were identified between SM vs. ST via RNA-seq. Fortunately for the sake of comparison, three DE-miRNAs (oar-miR-10a, oar-miR-143, oar-let-7c) were found in both studies [41]. To date, more studies on the miRNA expression profiles in sheep or goats are being published (with some also revealing profiles during different stages of wool production). For example, Liu et al. [42] investigated the miRNA expression profile of Chinese tan sheep hair follicles with different degrees of curliness, and reported the identification of 49 DE miRNAs. Interestingly, some of these DE-miRNAs (oar-miR-99a, oar-let-7c, and oar-miR-125b) have been associated with differing curliness in the hair follicles of both Hu sheep and Chinese tan sheep. Li et al. [43], on the other hand, explored 1354 DE miRNAs in the telogen skin between fine-wool sheep and Liaoning cashmere goats and reported the highest abundance of oar-miR-143 and oar-let-7c for these two groups. Similarly, Liu et al. [44] indicated that oar-let-7 and oar-miR-200 families might be the critical miRNAs in sheep hair follicle development.

Thus, in terms of available literature, many previously reported DE-miRNAs such as oar-miR-99a, oar-let-7c, oar-miR-125b, oar-miR-143, and oar-miR-200 were also present in our study. We, therefore, hypothesize that these miRNAs are likely to be involved in the growth and development of hair follicles in Hu sheep and influence the pattern formation of lambskin.

### 4.3. Functional Enrichment Analysis of DE miRNAs between SM vs. ST

To better understand the function of miRNAs, we performed functional annotation of miRNAs target genes. Research on hair follicle growth and development revealed that hair follicle development depended on a series of signaling molecules that participated in dermal and epithelial cell interactions—leading to the ordered proliferation and differentiation of the two cell populations, and ultimately resulting in the formation of intact hair follicles [45]. Fortunately, some target genes were enriched in an epithelial cell process, including the epithelial cell differentiation (GO:0030855), regulation of epithelial cell differentiation (GO:0030856), and epidermal growth factor catabolic process (GO:0007174), which were closely associated with hair follicle development.

The KEGG pathway analysis of target genes between SM vs. ST, some signaling pathways including MAPK signaling pathway, Ras signaling pathway, and PI3k-Akt signaling pathway were enriched. Recent studies suggested that the MAPK signaling pathway plays a positional role in hair follicle development and that activated MAPK signaling positively affected dermal papilla cell proliferation [38,46]. Additionally, the periodic changes of the important regulators in MAPK signaling pathway were shown to influence other key regulatory factors or signalings of Notch and FGF signaling pathways to regulate the development and differentiation of the hair follicle [47,48]. Surprisingly, activated PI3k-Akt signaling promoted the expression level of the crucial gene *TGF-β2* that forms part of the TGF-β signaling pathway thus indirectly affected hair regeneration [49]. Especially, activating PI3k-Akt signaling through TPA treatment could also increase the differentiation rate of hair follicle stem cells [37], whereas the Ras signaling pathway could also regulate hair follicle morphogenesis via acting as a role of regulating the expression of Shh signaling pathway [36].

### 4.4. Co-Expression Analysis of miRNA-mRNA

To explore the regulatory mechanism of miRNA, co-expression analyses of integrated miRNA-mRNA were performed to construct the interaction network by DE miRNAs and target genes. Regarding SM vs. ST, a total of 16 overlapped genes were targeted by 8 DE miRNAs.

Studies have revealed the consistent role of miR-125b in controlling the activity of various skin stem cells and its inhibition of stem cell proliferation by acting on the TGF-β/BMP signaling pathway [50,51,52,53]. It’s target gene, vitamin D receptor (*VDR*), could have a positive role in hair follicle differentiation [15], while in our study, miR-125b targeted *CD34* and *ZNF687*. *CD34* was the marker of hair follicle-associated-pluripotent stem cells, regenerating part of hair follicle [54]. *ZNF687*, on the other hand, is a member of the zinc finger family and zinc finger proteins (structural proteins) have been described to affect the hair follicle cycle. Among them, the zinc finger transcription factor Trps1 is specifically expressed in the mesenchymal cell nucleus during hair follicle morphogenesis [55,56] and *ZNF687* may be similarly involved [57]. MiR-200b was predicted to target 14 genes, including *MAP4K5*, *CTNND2*, *PAK3*, *SLC4A4*, *ZNF76*, *ZNF831*, etc. Previously, the miR-200 family has been reported as being highly expressed in epithelial cells where they contribute to the regulation of proliferation in hair morphogenesis [58]. As such, there is a possibility that its target genes may also be involved in hair follicle morphogenesis. It has also been found that miR-29a/b1 could target *Bmpr1a* and *Lrb6* to inhibit hair follicle stem cell differentiation via suppressing BMP and WNT signaling [59]. Considering that *NAV3* was the target gene of miR-29a, the role of *NAV3* in hair follicle morphogenesis was also worth exploring. In addition, miR-431 targeted *CD34*, *FGF12*, and *ZNF831*, which might be participated in hair follicle morphogenesis [60,61]. Overall, these key miRNAs may therefore be either directly or indirectly associated with hair follicle activity, and further research is needed to explore their function(s) regarding wool curvature.

In summary, the current study provided a Hu sheep hair follicle miRNA expression profile and unraveled its miRNA-mRNA interaction network. Several DE miRNAs, including miR-125b, miR-10a, miR-10b, miR-200b, miR-143, miR-29b, etc., were identified while miRNAs-mRNAs interaction relationships, which may be involved in hair follicle morphogenesis (miR-200b targeting *MAP4K5*, miR-125b targeting *CD34*, and miR-431 targeting *FGF12*), were highlighted. These results will provide a great basis for further exploration of wool curvature—such as the use of molecular breeding to produce new strains of Hu sheep with high-quality lambskin.

## Data Availability

The sequence data from this article have been submitted to SRA with the bioproject numbers PRJNA647222 and PRJNA632702.

## Supplementary Materials

The following supporting information can be downloaded at: https://www.mdpi.com/article/10.3390/genes13020342/s1, Figure S1: Statistics analysis of miRNA chromosome distribution; Figure S2: Pearson correlation between samples; Table S1: Primers information of miRNAs and mRNAs; Table S2: Overview of the quality control of miRNA reads generated from hair follicle tissues; Table S3: DE miRNAs from SM vs. ST in hair follicle; Table S4: Overview of the quality control of mRNA reads generated from hair follicle tissues; Table S5: DE mRNAs from SM vs. ST in hair follicle; Table S6: GO enrichment analysis of target genes; Table S7: KEGG enrichment analysis of target genes; Table S8: Target genes prediction analysis of DE miRNAs.
