# Supplementary figures and images for: Integrated Hair Follicle Profiles of microRNAs and mRNAs to Reveal the Pattern Formation of Hu Sheep Lambskin
Source: Genes (Basel). 2022 Feb 14;13(2):342. doi: 10.3390/genes13020342 (PMC8872417; doi:10.3390/genes13020342)

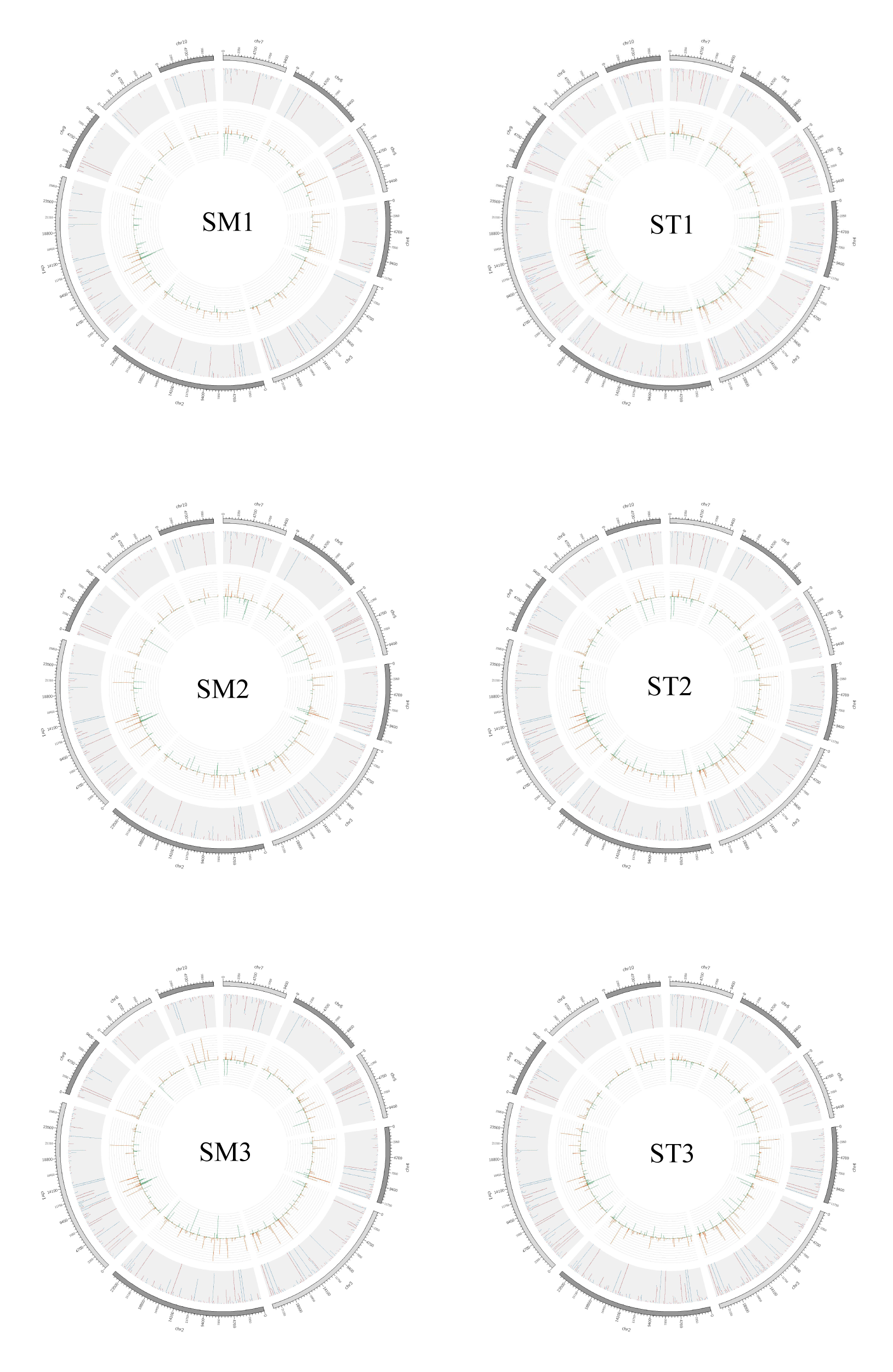

Supplement: Supplementary file 1 [file genes-13-00342-s001.zip › Supplementary Figure S1.tif]

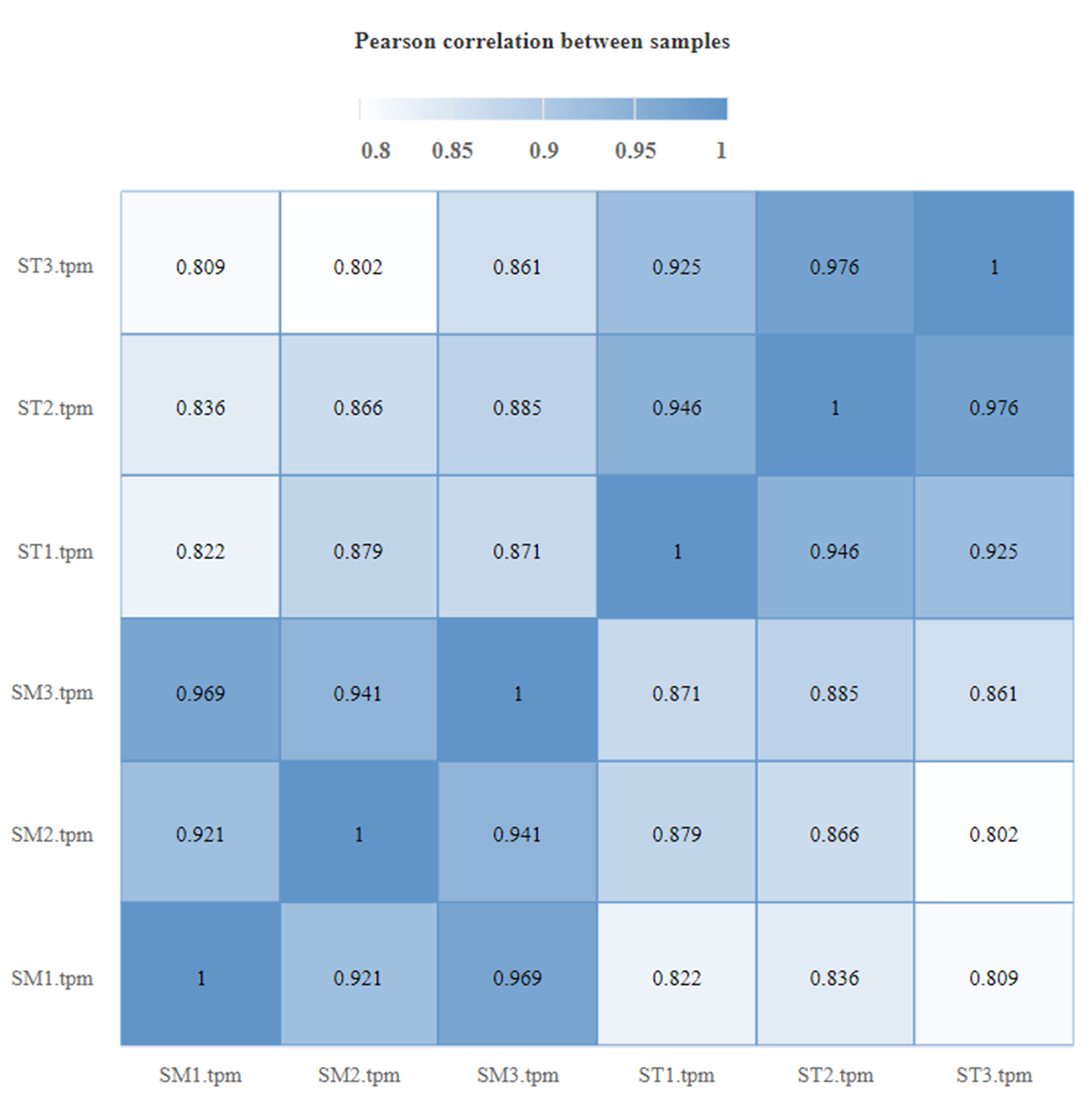

Supplement: Supplementary file 1 [file genes-13-00342-s001.zip › Supplementary Figure S2.tif]
